# Platelet-Derived Growth Factor (PDGF)/PDGF Receptors (PDGFR) Axis as Target for Antitumor and Antiangiogenic Therapy

**DOI:** 10.3390/ph3030572

**Published:** 2010-03-11

**Authors:** Marius Raica, Anca Maria Cimpean

**Affiliations:** Department of Histology and Molecular Pathology, “Victor Babes” University of Medicine and Pharmacy, Pta Eftimie Murgu 2, 300041 Timisoara, Romania; Email: acimpeanu@umft.ro (A.M.C.)

**Keywords:** angiogenesis, antiangiogenic therapy, antitumor therapy, platelet-derived growth factor (PDGF), platelet-derived growth factor receptor (PDGFR)

## Abstract

Angiogenesis in normal and pathological conditions is a multi-step process governed by positive and negative endogenous regulators. Many growth factors are involved in different steps of angiogenesis, like vascular endothelial growth factors (VEGF), fibroblast growth factor (FGF)-2 or platelet-derived growth factors (PDGF). From these, VEGF and FGF-2 were extensively investigated and it was shown that they significantly contribute to the induction and progression of angiogenesis. A lot of evidence has been accumulated in last 10 years that supports the contribution of PDGF/PDGFR axis in developing angiogenesis in both normal and tumoral conditions. The crucial role of PDGF-B and PDGFR-β in angiogenesis has been demonstrated by gene targeting experiments, and their expression correlates with increased vascularity and maturation of the vascular wall. PDGF and their receptors were identified in a large variety of human tumor cells. In experimental models it was shown that inhibition of PDGF reduces interstitial fluid pressure in tumors and enhances the effect of chemotherapy. PDGFR have been involved in the cardiovascular development and their loss leads to a disruption in yolk sac blood vessels development. PDGFRβ expression by pericytes is necessary for their recruitment and integration in the wall of tumor vessels. Endothelial cells of tumor-associated blood vessels can express PDGFR. Based on these data, it was suggested the potential benefit of targeting PDGFR in the treatment of solid tumors. The molecular mechanisms of PDGF/PDGFR-mediated angiogenesis are not fully understood, but it was shown that tyrosine kinase inhibitors reduce tumor growth and angiogenesis in experimental xenograft models, and recent data demonstrated their efficacy in chemoresistant tumors. The *in vivo* effects of PDGFR inhibitors are more complex, based on the cross-talk with other angiogenic factors. In this review, we summarize data regarding the mechanisms and significance of PDGF/PDGFR expression in normal conditions and tumors, focusing on this axis as a potential target for antitumor and antiangiogenic therapy.

## 1. Introduction

Angiogenesis, the process of new blood vessel formation, plays a crucial role in many normal and pathological conditions. Since 1971, when Folkman hypothesized that the growth of malignant tumor is angiogenesis-dependent [1], there were accumulated a lot of evidences that support initial findings and moreover, it was shown that angiogenesis can be inhibited by specific molecules. Angiogenesis is a multi-step process regulated by proangiogenic factors and specific inhibitors. In normal tissues, there is equilibrium between endogenous stimulators and inhibitors of angiogenesis. In last two decades there were identified and characterized many endogenous pro- and antiangiogenic molecules (reviewed in [2] and [3]). 

Based on the present evidences, the most investigated and efficient angiogenic molecule is vascular endothelial growth factor (VEGF), characterized twenty years ago [4]. It was shown that VEGF strongly stimulates proliferation and migration of endothelial cells (ECs) in both normal and tumoral tissues [5,6]. VEGF secreted by normal and tumor cells induces angiogenesis by binding its specific receptors, VEGFR1 and VEGFR2. Even from early experimental studies, VEGF was thought to be a promising target for the antiangiogenic therapy and a humanized antibody against VEGF, bevacizumab, was the first antiangiogenic drug introduced in the clinical practice [7]. 

Besides VEGF, other growth factors have been shown to have a significant proangiogenic effect, like fibroblast growth factor (FGF), transforming growth factors, hepatocyte growth factor, angiopoietin-1, platelet-derived growth factors (PDGF) and others [reviewed by 7]. Although the molecular mechanisms of angiogenesis are relatively well characterized, the successive intervention and relationships between these growth factors during the angiogenic cascade is less understood. This could explain in part results of antiangiogenic therapy in human cancers that are not as well as expected by comparison with those reported in experimental models.

Although characterized many years ago, only in last years PDGFs were extensively investigated in the field of normal and tumor-associated angiogenesis. This could be explained by minimum two reasons: on one hand PDGF and specific receptors were found to be expressed by a large variety of normal and tumor cells, and on the other, the availability of specific inhibitors already used as drugs in some human neoplasms. In this review will be detailed the role of PDGF/PDGFR axis in normal and tumor angiogenesis, evaluating these molecules as potential targets for the antiangiogenic and antitumor therapy.

## 2. PDGF and PDGFR Family

In the mid 1970s, several groups demonstrated the existence of a major serum growth factor for fibroblasts, smooth muscle cells and glial cells derives from platelets [8,9,10]. This factor was called platelet-derived growth factor (PDGF) and was purified a few years later by Heldin *et al.* [11], and the same team discovered the PDGF receptor as a receptor tyrosine kinase [12]. 

PDGF is a 30 kDa dimer composed of an A- and/or B-chain, which are encoded by separate genes and regulated independently. Two additional genes were identified encoding PDGF-C and PDGF-D polypeptides [13,14]. Each chain is encoded by an individual gene located on chromosomes 7, 22, 4, and 11, respectively [15]. PDGF describes a heparin-binding family of polypeptide growth factors denoted A, B, C, and D. PDGF-C and –D are secreted as latent, inactive factors, and a protease that has not definitively identified, possibly tissue plasminogen activator, is required for their extracellular activation. All four PDGF chains contain a highly conserved growth factor domain of approximately 100 amino acids that is also found in the VEGF family. Until now, five dimeric compositions have been identified: PDGF-AA, -BB, -AB, CC, and –DD [16]. PDGF target a broad spectrum of mesoderm-derived cells, like fibroblasts, pericytes, smooth muscle cells, glial cells or mesangial cells [17]. The PDGF isoforms bind two distinct class III receptor tyrosine kinases, PDGFRα and PDGFRβ. Binding of the ligand leads to autophosphorylation of the receptors on tyrosine residues and this event induces activation of several signaling molecules [18].

The individual PDGF chains have different affinities for the two receptors. PDGFRα has high affinity for PDGF-A, -B, and -C, whereas PDGFRβ has high affinity for PDGF-B and –D. These interactions can be demonstrated *in vitro*, but it is not known if all are effective *in vivo* [19]. Ligand-binding to receptors induces receptor dimerization, which leads to activation of the intrinsic tyrosine kinase domain and subsequent recruitment of SH-2-domain-containing signaling proteins [20]. Finally, activation of these pathways leads to cellular responses, like proliferation and migration.

Expression of activated p21Ras in cells influences PDGFRβ signaling at multiple levels. Two separate mechanisms are taken into account for defective PDGFRβ signaling: transcriptional down-regulation of PDGFRβ expression and inhibition of ligand-induced PDGFRβ by a factor of the cell membrane of p21Ras-expressing fibroblasts [21]. Reversion of the cell phenotype results in the recovery of the PDGFRβ kinase activity. Disruption of the fibroblast cytoskeleton leads to the loss of PDGFRβ function. 

The minimal promoter for the human PDGF-B gene comprises – 100 bp and some important transcription factors have been shown to interact with distinct sites in this region. Sp1 was the first endogenous nuclear factor demonstrated to bind the PDGF-B promoter – 30 bp of the TATA box, and this interaction mediates basal PDGF-B gene expression in endothelial cells (ECs) and smooth muscle cells [22,23]. Phosphorylation of Sp1 transcription factor mediates the inducible expression of PDGF-B-chain gene *via* atypical protein kinase C-ξ [24].

PDGF are major mitogens for many cell types of mesenchymal origin and for some cells that are neuroectodermal in origin, like oligodendrocytes. PDGF have chemoattractant properties and have been involved in bone formation, erythropoiesis, wound healing and angiogenesis [25], and in the normal development of the kidney, brain, cardiovascular and respiratory systems [26]. A lot of evidences support the implication of PDGF in tumor growth and development of specific lesions from inflammatory diseases and atherosclerosis. During normal development, cell proliferation significantly increases as a consequence of PDGF overexpression and decreases in PDGF null mutants.

PDGF signals through two cell-surface tyrosine kinase receptors, PDGFRα and PDGFRβ, and induces angiogenesis by up-regulating VEGF production and modulating the proliferation and recruitment of perivascular cells [27]. The angiogenic activity of PDGF might not only be based on the increased VEGF-A production, because PDGF-B stimulation induces an increased ECs lineage commitment and restricted differentiation of hematopoietic precursors [18]. In knockout models it has been shown the critical role of PDGF-B and PDGFRβ signaling in the establishment of functional blood vessels by recruiting and stabilization of perivascular cells [28]. 

VEGF-A enhances endothelial PDGF-B expression, whereas FGF-2 enhances perivascular PDGFRβ expression [29]. Co-stimulation with VEGF and FGF-2 induced a significant perivascular cell recruitment *in vitro* and formation of functional vasculature *in vivo*. These effects are suppressed by PDGFRβ neutralizing antibodies, and also by exogenous PDGF-B, which indicates the importance of preservation of the periendothelial PDGF-B gradient.

Another function of PDGF is to regulate transcriptional activity of thrombomodulin in human vascular smooth muscle cells. Co-expression of PDGF-B and thrombomodulin was demonstrated in an experimental model of ligated carotid artery. In this process, PDGF-B upregulates the transcription factor E26 transformation specific sequence-1 (Ets-1) [30]. In resting perivascular cells and ECs, Ets-1 is expressed at low levels, and is induced by stimulation with PDGF-B. This process is inhibited by rapamycin, which was demonstrated to have antiangiogenic and antilymphangiogenic properties. Although these findings were done on human tissues (*in vitro*), the contribution of this process to normal and pathological angiogenesis is not known. 

Based on its basic properties, PDGF plays an important role in the wound healing, stimulating cell proliferation, migration and angiogenesis. This role is related to some specific molecule of the extracellular matrix, like collagens or heparin. In *in vitro* experiments, it has been shown recently that heparin improves the binding of PDGF to collagen, and the PDGF-heparin-collagen complex promotes proliferation of fibroblasts, cell migration and vascularization [31]. 

A relatively new member of the PDGF family is PDGF-C that is widely expressed in muscle tissue, but its functions *in vivo* remain poorly characterized. PDGF-C is expressed in actively angiogenic tissues, like placenta, ovary, some embryonic tissues, and tumors, and promotes angiogenesis *in vivo* in the mouse corneal and chick embryo chorioallantoic models [32]. In the developing chick embryo, PDGF-C induced sprouting of preexisting vessels and the angiogenic response is transduced by PDGFRαα and αβ. Angiogenesis in the corneal model, assessed by microvascular density (MVD) and vessels’ maturation, was virtually identical for PDGF-C, -AB and -B. In this condition, Cao *et al*. [32] suggested that PDGF-C activates PDGFRαα heterodimers.

In tumor angiogenesis there is a complex interplay between cancer cells, ECs and other stromal cells. PDGF/PDGFR axis seems to be crucial in this interaction and thus, it became an important target of novel antiangiogenic therapies.

## 3. PDGF/PDGFR and Vascular Development

PDGF and PDGFR play a crucial role in the normal development of various organs, like lung, intestine, kidney, skin and testis. An important contribution of PDGF was demonstrated in the development, recruitment and protection of glial cells [reviewed by 33,34]. Particularly, PDGF-B and its cognate receptor, PDGFRβ, are essential for the development of the cardiovascular system. All members of the PDGF family display potent angiogenic activity *in vivo*, and from this point of view, PDGF-B/PDGFRβ axis was the most extensive evaluated. In the null mice it was shown that PDGF-B and PDGFRβ are critically involved in the vascular development.

The involvement of PDGF-B in the vascular development is an early event, and recently it was shown that PDGF-B stimulates differentiation of embryonic stem cells into ECs by calcium-mediated generation of reactive oxygen species [35]. *In vitro*, it was shown that PDGF-B can directly induce ECs proliferation, migration and tube formation, whereas PDGF-A lacks such effects. Moreover, PDGF stimulates not only ECs proliferation, but also VEGF secretion. Proangiogenic effects of different PDGF isoforms have been demonstrated *in vivo* in the chick embryo chorioallantoic membrane and in the mouse cornea pocket assay [32,36], and it was speculated that PDGF also are involved in tumor angiogenesis.

PDGF-B and PDGFRβ are mainly expressed in the developing vasculature in both normal and pathological conditions, including tumor angiogenesis. PDGF-B is produced by developing and quiescent ECs and PDGFRβ is expressed by perivascular cells and ECs [37]. When this paracrine signaling is disrupted, perivascular cells are not recruited and ECs proliferate irregularly, leading to improper vessel formation and hemorrhage [38]. During vasculogenesis and angiogenesis, PDGF act in concert with other proangiogenic factors to induce formation and stabilization of new vessels by recruitment of perivascular cells. 

PDGF-B is expressed by ECs and appears to signal only to PDGFRβ in perivascular cells. On the other hand, PDGF is expressed by ECs in a spatial-temporal pattern. PDGF-B expression by the endothelium is initially widespread and then becomes restricted to ECs at the sprouting tips. The tip cell was shown to be in close contact with pericytes and this could facilitate PDGF-B release [39]. Recruitment of pericytes is completely dependent on PDGF-B/PDGFRβ signaling, and this explains the near-complete loss of pericytes in organs such as the central nervous system in the absence of PDGF-B or PDGFRβ [34].

The role of PDGF/PDGFR in vascular development is supported by knockout experiments. PDGF-B and PDGFRβ knockout mice die perinatally from vascular defects found in many organs [40,41], despite the cause of this lethality has not been clearly identified. Deletion of either PDGFRα or PDGFRβ caused no overt vascular defects, but loss of both receptors led to a disruption in yolk sac blood vessels development in the transgenic mouse [42]. It was found that PDGFR expression in the yolk sac mesothelium is essential for blood vessel development through extracellular matrix deposition to promote vascular remodeling. This findings support the crucial contribution of PDGF signaling in vessel growth and the spectrum of its functions is broader than once thought.

The most important function of PDGF-B during development is to promote perivascular cells recruitment during angiogenesis [43] and is more strongly expressed in arterial than in the venous endothelium [44]. In the absence of PDGF-B the number of perivascular cells in the small blood vessels is reduced and their rate of proliferation is slower. In an experimental model it was shown that PDGFRβ are localized on sprouts and cords/tubes of angiogenic ECs, and their expression increased with cords/tubes formation. Neutralization of PDGF-B in human serum with anti-PDGF-B antibody reduced cord and tube formation in aortic endothelial cell culture [45].

In a recent study it was shown that synectin plays a role in the maturation of the arterial vessels and synectin deficiency induced increased degradation of PDGF in the arterial ECs and reduced perivascular cells recruitment [46]. However, no data are available about a similar role of synectin in tumor-associated blood vessels, and therefore, further studies are necessary to characterize a new potential target for antiangiogenic therapy.

Thus, PDGF-B plays a critical role in the maintenance of vascular stability through the attraction of perivascular cells expressing PDGFRβ. Stimulation of PDGFRβ with PDGF-B induced sprouting vasculogenesis in differentiating embryonic stem cells and accelerates the differentiation of ECs [47]. Increased PDGFRβ activity is associated with overexpression of VEGF-A and VEGFR-2, and results in increased sprouting, pericyte coating and vessel formation [18].

## 4. PDGF are Differentially Expressed by Normal Tissues

PDGF are expressed by a large variety of normal human tissues and organs. The highest expression of PDGF-A is found in the heart, skeletal muscle and pancreas. PDGF-B is expressed with the highest amounts in the heart and placenta, and moderate levels in other organs. PDGF-C is expressed with higher levels in the heart, kidney, adrenal gland, and pancreas, and with low levels in liver and ovary. No expression of PDGF-C can be detected in spleen or colon. The highest expression of PDGF-D was found in the heart, pancreas, and ovary and no detectable expression in the brain, lung and skeletal muscle [reviewed by 15].

**Table 1:** Distribution of PDGF and PDGFR in normal human tissues.

Tissue	PDGF-A	PDGFRα	PDGF-B	PDGFRβ
Blood vessel	-	-	Endothelium	Perivascular cells
Heart	Muscle cells	Mesenchyme	Muscle cells Endothelium	Perivascular cells
Lung	Epithelium	Mesenchyme	-	-
Kidney	Early nephron epithelium	Mesenchyme	Glomerular endothelium	Mesenchyme
Pancreas	Epithelium	Mesenchyme	-	-
Gut	Epithelium	Mesenchyme	-	-
Skin	Epidermis Hair follicle epithelium	Dermis	-	-
Nervous	Neurons Astrocytes	Astrocytes Oligodendrocyte precursors	Postnatal neurons	Postnatal neurons

**Figure 1:**
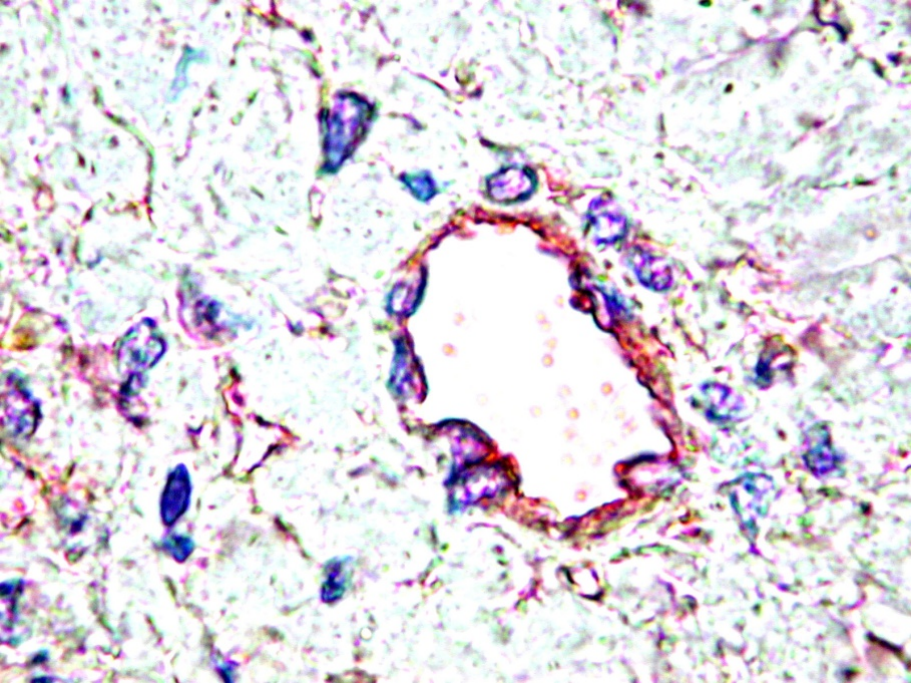
Double immunostaining for PDGF (red) and PDGFR-beta (brown, arrows). Original magnification, ×400.

Using a double immunostaining for PDGF-B and PDGFRβ, the final product of reaction for PDGF is found in the ECs, and the reaction is positive for PDGFRβ in the perivascular cells. Although PDGF-B is strongly expressed in the endothelium, only low levels of PDGFRβ expression can be detected in the perivascular cells of small vessels of the normal tissues (Figure 1). Different members of the PDGF family can be expressed in the same organ, but in different structures and this suggests potential functional differences. The differential expression of the four PDGF members was also documented in a panel of tumor cell lines [37].

## 5. PDGF and PDGFR in Cancer

The role of PDGF in carcinogenesis was initially demonstrated by the fact that *v-sis* oncogene encodes a PDGF-B-like protein. Both *v-sis* and its cellular counterpart *c-sis* transform cultured cells through an autocrine mechanism. In the last two decades, it was shown that PDGF and PDGFR are involved in human cancer development and progression through autocrine stimulation of tumor cell growth. In addition to the autocrine stimulation of tumor growth, PDGF signaling exerts paracrine stimulation on stromal cells and maybe the best certified example is tumor-associated angiogenesis. PDGF and PDGFR are involved in cancer by mutations that may lead to increased PDGF-levels or PDGFR activity, but retaining the structure and functions of these proteins [19]. The mode of action of PDGF and PDGFR in cancer development is largely autocrine and cell-autonomous as compared with the normal development where the mode of action is predominantly paracrine.

PDGF play minimum three roles that may lead to tumor development, including: (i) autocrine stimulation of cancer cells; (ii) stimulation of angiogenesis; (iii) control of tumor interstitial pressure. Blockade of autocrine stimulation of tumor growth by blocking PDGFR in cell lines and xenograft models showed consistent positive results in dermatofibrosarcoma protuberans, prostate cancer, ovarian cancer and gliomas (reviewed in [26]). In an experimental model it was shown that VEGF-null cells require PDGFRα for the recruitment of fibroblast in the tumor stroma. PDGF-A was demonstrated to be the major stromal fibroblast chemotactic factor produced by tumor cells and disrupting the paracrine signaling with PDGFRα significantly reduced tumor growth by inhibiting both tumor cells growth and angiogenesis [48]. 

PDGF and other growth factors, particularly VEGF, promote tumor-associated angiogenesis *via* autocrine and/or paracrine mechanisms as well as migration during tumor invasion. In an experimental model of glioma it was demonstrated that PDGF-B enhances angiogenesis by stimulating VEGF expression in tumor-associated ECs and by recruiting pericytes [49]. In the same model it was shown that PDGF-B enhanced the proliferation of both tumor cells and ECs. In human glioblastoma, PDGFRα is expressed by tumor cells and PDGFRβ is expressed by ECs of the newly-formed blood vessels [50]. The increased expression of PDGF and PDGFR in glial tumor cells and human tumors correlates with higher tumor grade. This indicate both autocrine and paracrine actions of PDGF in tumor progression and angiogenesis. Besides PDGF-B, PDGF-C and –D are expressed in brain tumors and play a critical role in maintaining cell transformation [51]. Recently, it has been shown that PDGF-C, expressed in 23 from 27 cases with glioblastoma, plays a role in the maturation of glioblastoma-associated blood vessels and its overexpression attenuates the response to anti-VEGF therapy *in vitro* [52]. 

The expression of PDGF and their receptors was demonstrated in a broad spectrum of human cancers, but for some of them, like prostate cancer or non-small lung cancer, their roles in tumor cell proliferation remain to be demonstrated. 

## 6. PDGF/PDGFR Are Expressed by Both Tumor and Stromal Cells

### 6.1. PDGF Expression in Tumor Cells

**Figure 2:**
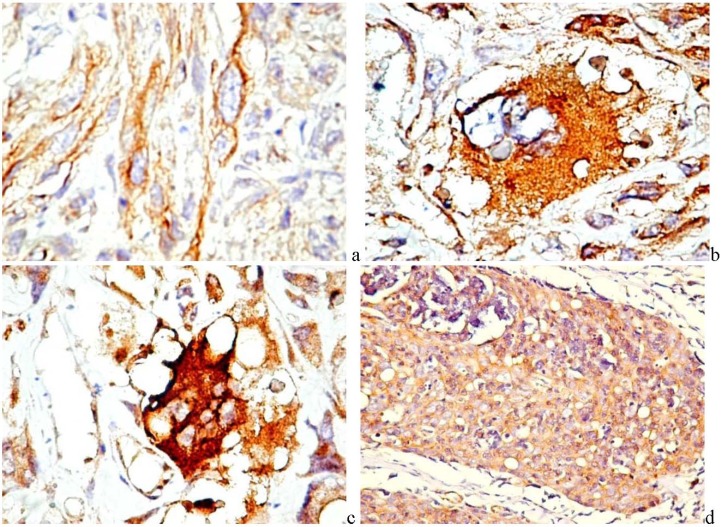
PDGF immunohistochemical expression in leiomyosarcoma (a, ×400), anaplastic renal cell carcinoma (b, ×400), malignant fibrous histiocytoma (c, ×400), and thymoma (d, ×200).

In carcinoma and melanoma, both tumor and stromal cells express PDGF. In glioma, fibrosarcoma and osteosarcoma, co-expression of PDGF and PDGFR by the tumor cells leads to an autocrine mechanism that drives carcinogenesis and tumor progression [51]. Using immunohistochemistry, PDGF was detected in tumor cells of various human tumors, like fibrosarcoma (Figure 2a), anaplastic renal cell carcinoma (Figure 2b), malignant fibrous histiocytoma (Figure 2c), or thymoma type B3 (Figure 2d). Usually, the expression has a cytoplasmic granular pattern, and besides tumor cells, it is also found in small blood vessels and scattered cells of the tumor stroma. Until now, no convincing correlations were found between PDGF expression by cancer cells and tumor grade, excepting for glial tumors.

The link between PDGF and tumor-associated angiogenesis is supported by its expression by tumor cells, and overexpression was found to be correlated with MVD and poor survival in a large variety of human cancers, including oral squamous cell carcinoma [53], pancreatic cancer [54], or early colon carcinoma [55]. It can be speculated that PDGF intervenes early in the colon carcinogenesis, as its expression was also noticed in tumor cells of mucosa-restricted neoplasms with stronger intensity than in the adjacent normal mucosa. In colon cancer, not only tumor cells can express PDGF, but also CD68-positive macrophages, mainly in cases with low VEGF expression, and PDGF-positive infiltrating cells strongly correlated with MVD [56]. 

In tumor transplantation models it was shown that the number and density of perivascular cells is higher when cancer cells overexpress PDGF-B [57]. Transplantation of a PDGF-B–secreting tumor in the PDGF ret mice induced perivascular cells detachment from the vessels’ wall. This suggests that perivascular cells may respond to ectopic sources of PDGF-B by migration away from the ECs [57]. Therefore, PDGF-B seems to be not only mitogenic for perivascular cell precursors, but also guide their migration during tumor angiogenesis.

PDGF is expressed in gliomas and sarcomas, which derive from cell types that are normally responsive to PDGF. Signaling through an autocrine PDGF/PDGFR loop is an early oncogenic event in gliomagenesis and the increased expression of PDGF-A and -B correlates with the degree of malignancy [58]. The overexpression of PDGF-B in glioma results in tumors with short latency, large area of necrosis, and angiogenesis, and PDGFRβ signaling is required for the maintenance of these characters [59]. This is supported by the treatment of experimental-induced glioma with PDGFR inhibitors that reverse the tumor histology to a lower grade.

**Figure 3:**
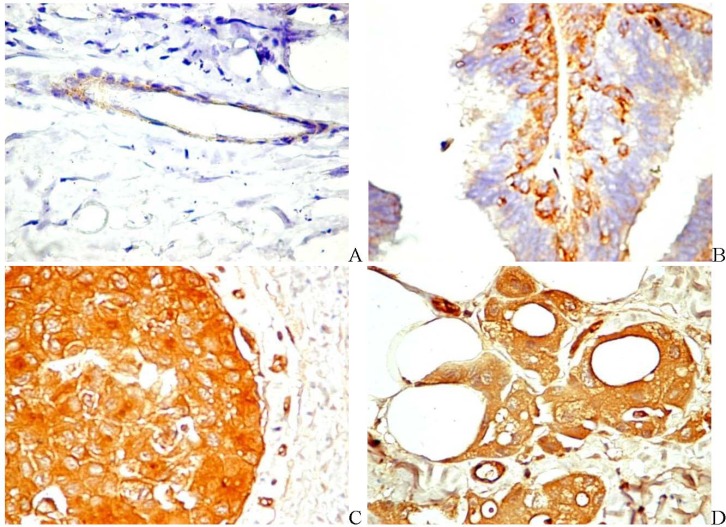
Immunohistochemical expression of PDGF in the normal mammary tissue (a), atypical intraductal papilloma (b), ductal in situ carcinoma (c), and invasive breast carcinoma (d). Original magnification A-D, ×400).

PDGF produced by prostate cancer cells induces the expression of PDGFR on tumor-associated ECs and activates PDGFR by a paracrine mechanism [60]. PDGF mRNA detected by semi-quantitative reverse transcription-polymerase significantly correlated with MVD of aggressive endometrial carcinoma [61]. This suggests that PDGF, together with VEGF, contribute to the aggressive potential of the tumor through the induction of angiogenesis. We found a particular mode of PDGF distribution in tumors of the breast. The immunohistochemical expression of PDGF gradually increases from the normal tissue to invasive carcinoma. As an example, in the normal mammary tissue, PDGF is usually detected in the perivascular cells (Figure 3a) and with low levels in myoepithelial cells. In the intraductal atypical papilloma, myoepithelial cells become prominent and PDGF is also expressed by ECs (Figure 3b). Almost all epithelial cells of the intraductal carcinoma in situ are intensely stained (Figure 3c), and the strong, granular cytoplasmic pattern is preserved in invasive carcinoma (Figure 3d) *(unpublished data)*.

Taken together, these data indicate that PDGFR overexpression is an independent poor prognostic factor. Data regarding PDGF-A/PDGFRα axis are controversial, as some authors reported a direct antiangiogenic effect of these molecules by inhibiting angiogenic properties of FGF-2, opposite to PDGF-B/PDGFRβ pathway that is proangiogenic [62]. Paradoxically, PDGF and probably other vascular remodeling factors could play a role in the normalization of tumor vasculature through perivascular cells recruitment, which might increase chemotherapeutic drug delivery. This effect needs further investigation because it is in part in conflict with the inhibition of PDGF/PDGFR axis disruption as anticancer strategy.

### 6.2. PDGFR in Experimental and Human Tumors

Aberrant signaling through PDGF/PDGFR pathway is involved in neoplastic transformation and tumor progression of a variety of cancers. An early oncogenic event in some malignant tumors, like glioma or prostate cancer, seems to be the establishment of the PDGF-autocrine loop. This is supported by the blockade of the PDGF/PDGFR pathway that results in growth inhibition and reversion of the transformed phenotype of glioma cell lines. However, co-expression of PDGF and PDGFR was demonstrated in glioma tumor cells and in soft-tissue sarcoma. PDGFR are expressed in tumor cells and stromal cells of neoplastic tissues. PDGFR have been shown to play a critical role in tumor progression as a part of the group of receptors expressed on the membrane of cancer cells. Particularly, PDGFR were found in breast carcinomas with aggressive behavior [63]. 

PDGFRβ is usually undetectable in quiescent ECs, but it is expressed by ECs of several human tumors, and capillaries are frequently surrounded by PDGFRβ-positive perivascular cells. Whereas PDGFRβ expression is common on tumor-associated pericytes, its expression on tumor-associated ECs appears to be more restricted. Overexpression of PDGFRβ has been demonstrated by in situ hybridization in the proliferative endothelium of gliomas [64]. In experimental mouse models it was shown that PDGFR-inhibitor imatinib associated to chemotherapy enhanced the therapeutic response, and tumor regression was associated with increased ECs apoptosis [60].

PDGFR are expressed in 50 to 70% of ovarian tumors and are activated in cancer cells through paracrine and autocrine mechanisms. Besides cancer cells, PDGFR are also expressed by fibroblasts, pericytes and ECs of the tumor stroma [65]. Thus, PDGFR inhibitor agents have direct inhibitory effects on tumor cells growth and indirect effects on the stroma, including increased delivery of chemotherapy into the tumor, and antiangiogenic effects induced in part by pericyte coverage disruption. Based on the significant role of PDGF and PDGFR in ovarian cancer growth, inhibition of PDGFR phosphorylation may be important in preventing the progressive growth.

**Figure 4:**
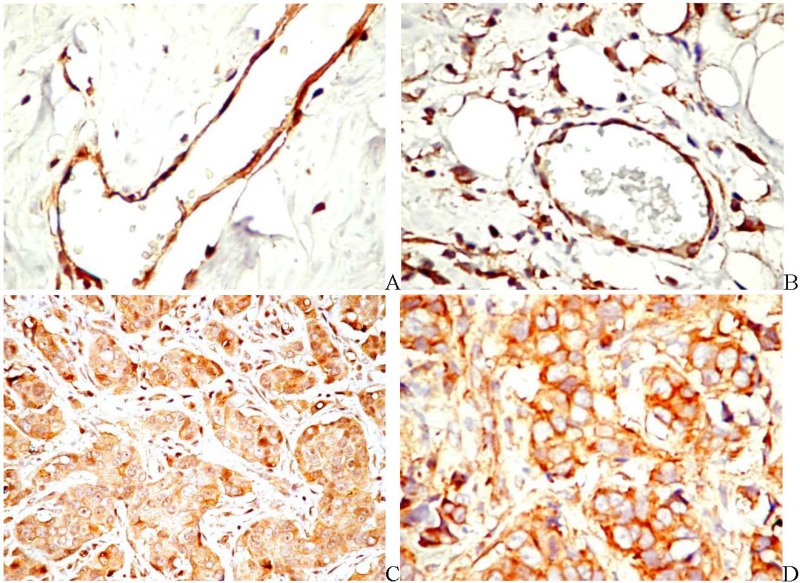
PDGFRα in the normal connective tissue (a, ×400). PDGFRβ in the tumor stroma (b, ×400). PDGFRα expression in both breast tumor cells and stromal cells (c, ×200). PDGFRβ expression in tumor cells from a breast invasive carcinoma (d, ×400).

PDGFRβ is expressed by both breast cancer cells and tumor-associated ECs in 69.7% of the cases [66]. It was shown that breast cancer cells induce PDGFRβ, but not PDGFRα expression in the adjacent ECs. The angiogenic effect of PDGFRβ was demonstrated by increased value of MVD, compared with PDGFRβ-negative specimens. Additionally, PDGFRβ was found in ECs growing in a bone metastasis breast cancer model [67]. In human specimens of breast cancer, both PDGFRα and β are expressed in the blood vessel wall. A differential expression was found in the stromal cells. PDGFRβ is expressed in a significant higher number of tumor-associated stromal cells than PDGFRα (Figure 4a and Figure 4b). PDGFR are also expressed by tumor cells, but the pattern of the positive reaction is again, different. PDGFRα is expressed in less than one third of the cases with moderate intensity and heterogeneous distribution (Figure 4c). PDGFRβ expression in tumor cells was found in more than two thirds of the cases, with strong and homogeneous distribution (Figure 4d). The most important aspect that comes from microscopic data is the increased expression of PDGFR in invasive breast cancer in neoplastic cells, stromal cells and blood vessels, as compared with the normal tissue and premalignant conditions *(unpublished data)*. These data indirectly support the link between tumor progression and angiogenesis induced by the PDGF signaling. Similar data were found in patients with prostate cancer, where PDGFR expression was associated with tumor progression and overexpression was found in the majority of bone metastasis [78]. Taken together, these findings support the introduction of anti-PDGFRβ antiangiogenic therapy in cancers with PDGFRβ-positive ECs.

Some studies have shown that FGF-2 and hypoxia up-regulate PDGFR in the tumor vasculature, but the mechanism by which PDGFRβ is up-regulated by tumor cells needs further investigations. Experiments using anti-FGF-neutralizing antibodies and the correlation between PDGFRβ and HIF-1α should be performed to verify these hypotheses. Although PDGFRβ effects on angiogenesis were strongly documented, the mechanism of angiogenesis maintenance is not fully understood. This is suggested by the work of Tsusumi *et al*. [69], which found that PDGFRα is critical for the maintenance of angiogenic signals using VEGF and hepatocyte growth factor.

### 6.3. Targeting PDGF Signaling in the Tumor Stroma

Traditionally, the main targets for anticancer therapy are tumor cells, and more recently, the activated ECs that characterize tumor-associated angiogenesis. In last years, there were accumulated many data that support important roles of other cellular players in the development and progression of malignant tumors. Tumor stroma creates the microenvironment that promotes and maintains proliferation of neoplastic cells. PDGF is expressed by a variety of stromal cells, and particularly by fibroblasts. More than half of the lung cancers, colon cancers, breast cancer and melanomas, and one third of the prostate and ovarian cancers express PDGFRβ in the stromal cells. Fibroblasts have a prominent role in progression, growth and spreading of tumor cells. During tumor progression, fibroblasts are protected from the oxidative damage by tumor cells-secreted PDGF *via* the phosphoinositide 3-kinase pathway [70]. This data suggest that prosurvival signals initiated by tumor cells in the fibroblasts maybe a strategy of “stromal resistance”. PDGF-A was demonstrated to be a major fibroblast chemoattractant and mitogen in an experimental model of lung carcinoma. The levels of PDGFR strongly correlate with fibroblast infiltration in the tumor mass [71]. Stromal fibroblasts also stimulate tumor angiogenesis by providing additional VEGF. This suggests that targeting stromal cells may be effective in treating certain types of solid tumors.

Moreover, tumor-associated fibroblasts express PDGFR, and seem to play a crucial role in both proliferation of tumor cells and angiogenesis. In a mouse model of cervical carcinogenesis, it was shown that targeting in particular tumor-associated fibroblasts with the PDGFR inhibitor imatinib mesylate, results in slowed progression of premalignant lesions and impaired growth of invasive carcinoma [72]. Similar effects were obtained by neutralizing anti-PDGFR antibodies and results were explained by suppression of FGF-2 and FGF-7, and supported by decreased proliferation of tumor cells and inhibition of angiogenesis. 

Fibroblasts are thought to promote tumor growth in part through stimulation of angiogenesis. In a recent study, it was shown that PDGF-C is up-regulated in tumor-associated fibroblasts from tumors refractory to anti-VEGF treatment [73]. PDGF-C stimulates migration of ECs and induces angiogenesis in the chick embryo chorioallantoic membrane [32]. PDGF-C neutralizing antibodies suppressed angiogenesis induced by tumor-associated fibroblasts *in vivo*, and are additive with anti-VEGF antibodies. Altogether, these data suggest that a combination of VEGF antagonists with PDGFR inhibitors might be an effective anticancer strategy [74], but such an approach could be associated with marked toxicity.

Another particular aspect of PDGF/PDGFR effects on tumor stroma is related to the regulation of interstitial pressure. Changes in the interstitial pressure can modify the drug uptake by tumor cells. Targeting PDGFR in the tumor stroma becomes a novel strategy to increase the efficacy of chemotherapy through a tumor-selective increase in drug uptake. These experimental studies need to be validated in clinical trials.

## 7. PDGF and Tumor Experimental Models

Many experimental models were designed in both *in vitro* and *in vivo* to demonstrate the contribution of PDGF/PDGFR axis in tumor progression, the link with tumor-associated angiogenesis, and the effects of specific inhibitors. Increased levels of PDGF were found in the serum and tumor cells of patients with squamous cell carcinoma of the head and neck [75]. Treatment of these cell lines with imatinib resulted in reduced secretion of PDGF and VEGF that supports the connection between PDGF and VEGF pathways in squamous cell carcinoma. In the B16 mouse melanoma model, production of PDGF-B or PDGF-D by tumor cells is associated with an increase in the number of pericytes within the tumor and increased tumor growth rate, but without a significant increase in MVD [76]. This suggests that the increased pericyte coverage affected tumor vasculature in a functional rather than quantitative manner. PDGF-B expression increases tumor vasculature of B16 melanoma cell-induced tumor and PDGFRβ accelerates tumor growth. Suzuki *et al*. [77] have shown an increased vessel area and surface in mouse carrying activated PDGFRβ, but not an increase in the vessel count. Taken together, these studies demonstrate the critical role of PDGF in the recruitment of pericytes during tumor angiogenesis.

Campbell *et al.* [78] developed a PDGF-C transgenic mouse model that mimics human liver carcinogenesis. Overexpression of PDGF-C resulted in liver fibrosis and development of dysplastic lesions and angiogenesis, followed by progression to hepatocellular carcinoma. The treatment with the tyrosine kinase inhibitor imatinib, decreased the proliferation of non-parenchyma cells *in vivo* and *in vitro*, and correlated with lower levels of PDGFRα. This finding suggests that imatinib could be efficient in the treatment of hepatocellular carcinoma, particularly in the presence of an active angiogenesis. Blocking the PDGFR signaling, in a transgenic mouse model of pancreatic islet carcinogenesis (Rip1Tag2) with the receptor tyrosine kinase inhibitor SU6668 caused regression of blood vessels, which was due to the detachment of perivascular cells from tumor vessels, and restricted tumor growth [67]. Xenografts of glial tumor cells have also been used to study and to validate the importance of autocrine PDGF signaling in brain tumor progression. Tumor xenografts were responsive to treatment with imatinib, and based on these data, there were initiated clinical trials in patients with glial tumors.

The intervention of PDGF-B in tumor growth and angiogenesis seems to be organ- and tumor type-dependent. In a murine model of fibrosarcoma, it was found that PDGF-B and FGF-2 synergistically promote tumor angiogenesis and metastasis, but no response was achieved using PDGF-B alone [79]. It was suggested that FGF-2 acts as a sensitizer for ECs to respond to PDGF-B signaling, and this feeds back to perivascular cells to enhance their response to FGF-2 stimulation. These events lead finally to the development of new vessels, accelerated tumor growth and metastasis. 

In an experimental model of skin carcinogenesis it was demonstrated that fibroblast are essential for mediating transient angiogenesis and epithelial proliferation and PDGF-B treatment induced an initial up-regulation of VEGF, followed by a drastic VEGF down-regulation coincident with myofibroblast differentiation [80]. The time-dependent effects of PDGF-B on fibroblasts is supported by an initial recruitment of proliferating cells and induction of angiogenesis, but followed by a delayed maturation of newly-formed blood vessels. The mechanisms by which PDGF-B induces angiogenesis is not fully understood, but it can be speculated that the main effects are achieved directly by PDGFRβ activation or/and indirectly by PDGF-secreting fibroblasts that enhances the amount of stromal VEGF.

Additionally, it was shown that imatinib inhibited the growth of neuroblastoma cells *in vitro* and *in vivo*, process associated with suppression of PDGFR and c-kit phosphorylation and inhibition of VEGF expression [81]. Oral administration of SU11657 (Sugen), a selective multitargeted tyrosine kinase inhibitor, to athymic mice resulted in significant inhibition of human neuroblastoma xenografts and reduced tumor angiogenesis by inhibiting PDGFRβ and VEGFR-2 [82]. A selective PDGFR inhibitor, CP-673,451, also inhibits tumor PDGFRβ phosphorylation, selectively inhibits PDGF-B-induced angiogenesis *in vivo*, and caused significant tumor growth inhibition in human xenograft models [83]. A neutralizing antibody produced against mouse PDGFRβ enhanced the anti-tumor and antiangiogenic effects of an anti-VEGFR-2 antibody, and administration of both resulted in tumor regression and inhibition of angiogenesis in xenograft models [84]. These experimental data support to use PDGFRβ antagonists in combination with other antitumor and/or antiangiogenic agents in the treatment of a variety of cancers.

## 8. PDGF/PDGFR Axis and Tumor Prognosis

The progressive growth of many human carcinomas, including those of prostate [60], ovary [85], lung [86], stomach [87], breast [88], and melanoma [89], has been associated with expression of PDGFR or PDGFR ant its ligand. The overexpression of PDGFR was associated with a reduced overall survival. In non-small lung cell carcinoma, PDGF-B, -C and PDGFRα are negative indicators for disease-free survival. On the other hand, high expression of PDGF-A, -B, -D and PDGFRα in tumor stroma are correlated with good prognosis [90]. The expression of PDGFR was found to be decreased in human metastatic melanoma as compared with the normal skin and benign lesions, and it was suggested that loss of PDGFR may represent a way to select more aggressive clones that sustain melanoma progression. This is also supported by the inhibition of tumor growth and angiogenesis by PDGFRα in an experimental model of malignant melanoma [91]. Although experimental studies demonstrated the involvement of PDGF/PDGFR pathway in melanoma progression and metastasis, this issue deserves further investigations, because no clinical benefits were found in clinical trials with PDGFR inhibitors in metastatic melanoma [92]. Based on these data, it can be speculated a complex cellular crosstalk between tumor cells and ECs when targeting PDGF/PDGFR axis. Although PDGF and its receptors are generally thought to be good indicators of prognosis, there are still controversial and lack of data in some particular human tumor, as shown in Table 2.

**Table 2:** Expression of PDGF and PDGFR by tumor cells and the relationship with tumor progression and metastasis.

Tumor	PDGF-A	PDGF-B	PDGFRα	PDGFRβ	Correlation with	Ref
Nephroblastoma (n=62)	50%	ND	55%	ND	Progression	[93]
NSCLC (n=335)	98%	100%	98%	98%	LNM	[94]
Hodgkin lymphoma (n=65)	ND	ND	95%	ND	ND	[95]
Non-Hodgkin lymphoma (n=50)	ND	ND	48%	ND	No correlation	[96]
Recurrent ovarian cancer (n=44)	88.4%	69.8%	90.9%	88.6%	No response to imatinib therapy	[97]
Colorectal carcinoma (n=60)	ND	60%	ND	ND	Vascular invasion	[98]
Osteosarcoma (n=54)	80.4%	75.4%	79.6%	86%	DFS for PDGF-A/PDGFRα	[99]
Cervical adenosquamous carcinoma (n=27)	ND	ND	100%	ND	NS	[100]

Legend: NSCLC, non-small-cell lung carcinoma; LNM, lymph node metastasis; ND, not determined; NS, not significant; DSF, disease free survival.

PDGF-D is a newly characterized growth factor that regulates cell proliferation, transformation, invasion, and angiogenesis by activating its cognate receptor PDGFRβ. The functions of PDGF-D during the progression of solid tumors are largely unknown but there were accumulated evidences that support its involvement in the local progression and development of metastasis [101]. Taken together, these data indicate that PDGFR expression by tumor cells could be an individual prognostic factor in a variety of malignant tumors. 

## 9. The Role of PDGF/PDGFR in Lymphangiogenesis

Intratumoral and peritumoral lymphatic vessels facilitate the spread of tumor cells to regional lymph nodes in some of the most common cancer types, like breast cancer, colorectal carcinoma or malignant melanoma. Therefore, understanding the molecular mechanisms that control lymphangiogenesis could be an important step in developing therapeutic agents for prevention and treatment of cancer metastasis. The evaluation of lymphangiogenesis in cancer is strongly focused on VEGF-C and VEGF-D that interact with the specific receptor VEGFR-3. These two growth factors enhance lymphatic metastasis when expressed at high levels by cancer cells. Besides VEGF-C and -D, other growth factors that may contribute to lymphangiogenesis were detected in tumor cells. PDGF-B expression has been found in breast cancer [102], and can be a chemoattractant for primary lymphatic endothelial cells. 

In a mouse corneal lymphangiogenesis model, it was shown that PDGF-B induces formation of lymphatic vessels at day 5 after the implantation [103]. The number of lymphatic vessels induced by PDGF-A was significantly lower than that induced by PDGF-B, and this finding suggests the role of PDGFRβ in induction of lymphangiogenesis. The direct role of PDGF-B in the induction of lymphangiogenesis is supported by the blockage of VEGF-C/-D, and VEGFR-3, which did not inhibit PDGF-B-induced lymphangiogenesis [103]. These data also suggest that PDGF-B may modulate postnatal remodeling of lymphatic vessels. PDGF-B was demonstrated to stimulate migration of lymphatic endothelial cells, and PDGFRα and β were detected in newly formed lymphatic vessels [104]. Additional data come from the implantation of PDGF-B-expressing tumor cells into the syngeneic mice and resulted in accelerated tumor growth [103]. In this experiment, high density of lymphatics was found in PDGF-B-expressing tumors, associated with high incidence of regional lymph node metastasis.

Based on these findings, it can be speculated on one hand that PDGF-B is a direct lymphangiogenic factor, and on the other, that blockage of PDGFR activation inhibits PDGF-B-induced lymphangiogenesis and limits tumor growth and lymph node metastasis. These data should be validated on other genetic models, including PDGF/PDGFR knockout mice for potential lymphangiogenic defects. Although no studies have yet demonstrated antitumor effects targeting PDGF alone, the effect of PDGF on perivascular cells and tumor stroma points to the possibility that PDGF may cooperate with other angiogenic molecules to support both angiogenesis and lymphangiogenesis.

## 10. PDGF/PDGFR Axis and Therapy

Overexpression of PDGF and PDGFR was reported in many human malignancies and some cancer patients have high serum levels of PDGF. Elevated levels of PDGF and PDGFR in cancer patients correlate with poor response to chemotherapy and shorter survival. Tumor cells and tumor-associated ECs express activated PDGFR. Inhibiting phosphorylation of the PDGFR with a PDGFR tyrosine kinase inhibitor became a therapeutic strategy, largely investigated in last years. Inhibition of PDGFR activation may decrease cell proliferation and increase the rate of apoptosis. PDGF antagonists include neutralizing antibodies against ligands or receptors, inhibitors of receptor dimerization, and low-molecular-weight compounds which act through competitive binding to the active site of the receptors [20]. Macromolecular compounds acting extracellularly have higher specificity but inferior pharmacological properties, as compared to the kinase inhibitors [20]. Targeted disruption of PDGF-B of PDGFRβ in mice results in ablation of pericytes and thus, these cells could be a target for the antiangiogenic therapy. Pericytes, like other perivascular and stromal cells, express PDGFRβ, and targeting this receptor can be beneficial even in the absence of its oncogenic form [67].

The best known inhibitor of PDGFR is imatinib mesylate, a small-molecule adenosine triphosphate analog. Imatinib has been evaluated in a number of malignancies, and complete or partial response was achieved in dermatofibrosarcoma protuberans, glioblastoma, prostate and ovarian cancer. Additionally, it was shown that imatinib inhibits the proliferation of neuroectodermal tumors, like Ewing’s sarcoma or neuroblastoma [81]. On the other hand, imatinib as a single agent had no clinical effect in PDGFRβ-expressing advanced-stage breast cancer, did not changed the plasma levels of angiogenic molecules, and showed potential immunosuppressive effects [105]. This is in contrast with the expected results, because the overexpression of PDGFR is a common finding in the majority of invasive breast cancers in both tumor and stromal cells. 

There were accumulated data that suggest that such a strategy is useful especially in multidrug-resistant solid tumors. It was found that in human multidrug-resistant prostate cancer cell line, administration of the tyrosine kinase inhibitor imatinib, associated with paclitaxel, induces a decrease in the number of bone metastases. PDGFR phosphorylation was inhibited in both ECs and cancer cells, and this increased the apoptotic rate, decreased MVD, tumor size and lymph node metastases [106]. These data suggest that the main target for imatinib in the experimental model of prostate cancer is the endothelial cell. Consecutively, imatinib, sorafenib, dasatinib, sunitinib and neutralizing PDGFR antibodies are being investigated in clinical trials in patients with human cancer, like recurrent ovarian carcinoma [70] or prostate cancer [107]. Recently, it was shown that imatinib sensitizes chemoresistant glioma cells to cisplatin toxicity, depending on the Akt inactivation [108]. 

In many pathologic conditions and particularly in proliferative lesions, therapeutic inhibition of only one angiogenic factor fail to inhibit angiogenesis. In endometriosis lesions it has been shown that selective blockade of VEGF with tyrosine kinase inhibitor SU5416 (semaxanib) resulted in a slight decrease in MVD. In contrast, combined inhibition of VEGF, FGF and PDGF with SU5416 and SU6668 resulted in a marked inhibition of angiogenesis and blood vessel maturation [27]. The same effects were obtained by blocking VEGFR and PDGFR in a mouse model of pancreatic islet cell cancer with SU10944 and imatinib associated to metronomic chemotherapy. Using this regimen, 81% partial response was achieved and a prolonged median survival of the mice [109]. This is another proof that demonstrates that angiogenesis is not solely driven by VEGF, but by the crosstalk between many angiogenic factors. Such a therapeutic strategy needs further validation, because in a more recent study, using highly specific soluble receptors, Kuhnert *et al*. [110] suggested that additivity between VEGFR and PDGFRβ inhibition depends on the strength of VEGF blockade and appears minimal under conditions of maximal VEGF antagonism.

Evaluating the effects of tyrosine kinase inhibitors on ECs, in prostate cancer and glioblastoma *in vivo* and *in vitro*, Timke *et al*. [111] found enhanced apoptosis, reduced cell proliferation, and reduced migration of ECs and tube formation. More important, these effects were enhanced by additional irradiation to the dual antiangiogenic therapy (SU5416 and SU6668), with a significant delay in tumor growth. It was found that radiation induces an up-regulation of all four isoforms of PDGF that may explain in part the tumor escape from radiation damage. Addition of SU6668 reduces the paracrine radiation effect and contributes to a better antitumor effect. Thus, the combination of radiotherapy with antiangiogenic/antivascular therapy may become an interesting anticancer strategy and already entered the clinical trials. However, despite these data are convincing for prostate cancer and glioblastoma, in many other human solid tumors it is still unclear which combinations of signaling inhibitors would be most effective and which combination would benefit from the addition of radiotherapy.

Another tyrosine kinase-inhibitor, sunitinib, was recently shown to be effective in patients with metastatic renal cell carcinoma and increased the overall survival, but only 70% of the treated patients received a clinical benefit [112]. The efficacy in the control of the disease is even higher if sunitinib is associated to interferon-alpha therapy. Unfortunately, until now there are no specific serum markers with predictive value for the response to the treatment with tyrosine kinase-inhibitors in metastatic renal cell carcinoma.

The effects of tyrosine kinase-inhibitors on tumor growth and angiogenesis were investigated not only in solid tumors, but also in hematological malignancies. In a preclinical study it was shown that PDGF-B/PDGFRβ pathway promoted tumor growth and vessel sprouting in multiple myeloma, and dasatinib, a PDGFRβ/Src inhibitor delayed tumor growth and angiogenesis [113].

PDGFRβ has been identified as an important drug target in tumor therapy, based on the overexpression of PDGF-B by many solid tumors. It is still unclear whether inhibition of VEGF and PDGF together is more effective that inhibition of either one alone. In a recent experimental study on Rip-Tag2 tumors and Lewis lung carcinomas, Sennino *et al*. [114] demonstrated a differential expression of VEGF and PDGF and different effect on the microvasculature of specific inhibitors. This finding suggests that the response to angiogenic inhibitors could be determined by the cellular source and amount of angiogenic molecules, relationships between pericytes and ECs, and tumor phenotype.

Although tyrosine kinase inhibitors, and particularly imatinib, have been shown effective in gastrointestinal stromal tumors and myelomonocytic leukemia, no benefit was reported in clinical trials in a variety of advanced-stage human tumors, like metastatic breast cancer [105], metastatic melanoma [115], multiple myeloma [116], and hepatocellular carcinoma [117]. Therefore, it is a strong need to identify more effective combinations of tyrosine kinase inhibitors with other chemotherapeutic agents, and to identify new inhibitors of PDGF/PDGFR signaling. 

A lot of efforts were made in order to identify other specific molecule with inhibitory effects on the PDGF/PDGFR axis. A small molecule, GFB-111, which binds to PDGF, prevents it from binding to its receptor tyrosine kinase, blocks PDGF-induced phosphorylation, and in the nude mouse model showed significant inhibition of tumor growth and angiogenesis [119]. GFP-111 molecule was effective on glioblastoma cell lines, but not in medulloblastoma cells. The mechanism by which this molecule interfere tumor growth is not completely understood, but it was found a marked decrease of MVD. In a recent study, it has been shown that delphinidin, a major biologically active constituent of berries, rapidly inhibits activation of PDGFRβ in perivascular cells, suppresses vessels formation, migration of pericytes and capillary-like tubular structures formation in three-dimensional co-culture systems, and exerts an antitumor activity [120].

## 11. Perspectives

PDGF family includes peptide growth factors that signal through cell surface tyrosine kinase receptors and stimulate various cellular functions, like growth, differentiation, and proliferation. To date, PDGF and PDGFR expression was certified in a broad spectrum of human tumors and the autocrine growth stimulation is well documented. In addition, PDGF can regulate stromal cell through paracrine mechanism. Improved methods for detection of activated PDGFR would be more useful to characterize PDGF signaling in human tumors, and this could be useful to test PDGFR antagonist in clinical trials. 

The heterogeneity of angiogenesis in human tumors and the different ECs phenotype in different organs indicate that further investigations are needed to understand the interactions between tumor cells and ECs in cancer of various organs. In such a way, specific and optimal treatment regimens with targeting antivascular agents could be developed. There is increasing evidence that suggests a role of PDGFR antagonists in the treatment of cancer patients, mainly in specific tumors where the autocrine PDGFR stimulation is important. Combination of PDGFR antagonists with other targeted therapies, like VEGFR inhibitors and chemotherapy seems to be promising. However, clinical trials suggest that careful research is needed to balance between advantages and side effects of PDGFR inhibitors.

In last years, there were accumulated a lot of data that support the role of stromal cells in tumor progression and tumor-associated angiogenesis. It is possible that the phenotypic changes in tumor-associated stromal cells are enough stable for their use as targets of tumor growth inhibition. Isolation of distinct population of stromal cells and their characterization by gene analysis would give important information for their targeting approaches [120].

Critical protein targets that are known to be essential in the tumor growth must be identified and validated as anticancer therapy targets. Disruption the function of these targets must be developed and shown to selectively block the growth of tumor cells and angiogenesis. There is a strong need for more specific antibodies suitable for immunohistochemistry to detect activated PDGFR, and based on the expected impact on clinical decision a careful validation of their specificity will be required. High quality biomarker studies should be conducted to test the predictive value of these candidate biomarkers in clinical trials design. Certain biomarkers have not been tested in patients receiving PDGR inhibitors. It is important to develop combination antitumor regimens using predictive biomarkers in terms of dose and schedule. Further studies based on biomarkers research should provide data to support the realistic approach of antitumor and antiangiogenic therapy based on the PDGF/PDGFR inhibitors.
